# Radiomics of Contrast-Enhanced Computed Tomography: A Potential Biomarker for Pretreatment Prediction of the Response to *Bacillus* Calmette-Guerin Immunotherapy in Non-Muscle-Invasive Bladder Cancer

**DOI:** 10.3389/fcell.2022.814388

**Published:** 2022-02-25

**Authors:** Lei Ye, Yuntian Chen, Hui Xu, Zhaoxiang Wang, Haixia Li, Jin Qi, Jing Wang, Jin Yao, Jiaming Liu, Bin Song

**Affiliations:** ^1^ Department of Radiology, West China Hospital, Sichuan University, Chengdu, China; ^2^ Department of Urology, Institute of Urology, West China Hospital, Sichuan University, Chengdu, China; ^3^ Philips Healthcare, Chengdu, China; ^4^ University of Electronic Science and Technology of China, Chengdu, China

**Keywords:** BCG immunotherapy, NMF (nonnegative matrix factorization), NMIBC (non-muscle-invasive bladder cancer), CECT images, radiomics analysis

## Abstract

**Background:** *Bacillus* Calmette-Guerin (BCG) instillation is recommended postoperatively after transurethral resection of bladder cancer (TURBT) in patients with high-risk non-muscle-invasive bladder cancer (NMIBC). An accurate prediction model for the BCG response can help identify patients with NMIBC who may benefit from alternative therapy.

**Objective:** To investigate the value of computed tomography (CT) radiomics features in predicting the response to BCG instillation among patients with primary high-risk NMIBC.

**Methods:** Patients with pathologically confirmed high-risk NMIBC were retrospectively reviewed. Patients who underwent contrast-enhanced CT examination within one to 2 weeks before TURBT and received ≥5 BCG instillation treatments in two independent hospitals were enrolled. Patients with a routine follow-up of at least 1 year at the outpatient department were included in the final cohort. Radiomics features based on CT images were extracted from the tumor and its periphery in the training cohort, and a radiomics signature was built with recursive feature elimination. Selected features further underwent an unsupervised radiomics analysis using the newly introduced method, non-negative matrix factorization (NMF), to compute factor factorization decompositions of the radiomics matrix. Finally, a robust component, which was most associated with BCG failure in 1 year, was selected. The performance of the selected component was assessed and tested in an external validation cohort.

**Results:** Overall, 128 patients (training cohort, *n* = 104; external validation cohort, *n* = 24) were included, including 12 BCG failures in the training cohort and 11 failures in the validation cohort each. NMF revealed five components, of which component 3 was selected for the best discrimination of BCG failure; it had an area under the curve (AUC) of .79, sensitivity of .79, and specificity of .65 in the training set. In the external validation cohort, it achieved an AUC of .68, sensitivity of .73, and specificity of .69. Survival analysis showed that patients with higher component scores had poor recurrence-free survival (RFS) in both cohorts (C-index: training cohort, .69; validation cohort, .68).

**Conclusion:** The study suggested that radiomics components based on NMF might be a potential biomarker to predict BCG response and RFS after BCG treatment in patients with high-risk NMIBC.

## Introduction

Bladder cancer (BCa) is one of the most common cancers worldwide (Siegel et al., 2020). Based on the presence of muscular-invasiveness, BCa is pathologically categorized into muscular invasive BCa (MIBC) and non-muscular invasive BCa (NMIBC). Currently, the standard care for patients with NMIBC with a high-risk of recurrence is *Bacillus* Calmette-Guérin (BCG) instillation along with transurethral resection of bladder tumor (TURBT) (Babjuk et al., 2019). This therapy is effective in reducing recurrence and progression and increasing the survival of patients with high risk (Babjuk et al., 2019). However, approximately 40–60% of patients experience tumor recurrence within 2 years (Kamat and Sylvester, 2016). The earlier the tumor recurrence or BCG response is predicted, the better the patients’ chances of recieving new or alternative therapies because of the high recurrence rate after BCG treatment (Lotan et al., 2017).

However, no standard method has been established for predicting responses to BCG instillation. As the outcome of BCG instillation tends to vary across molecular characteristics, how to make a quantitative pretreatment prediction on the recurrence or progression after BCG treatment for better treatment planning is still a great challenge (Tran et al., 2020). Various biochemical indicators have been proposed to predict the responses to BCG in patients with high-risk NMIBC, including urinary and serum cytokine/chemokine profiles, and peripheral blood counts, such as eosinophils, neutrophils, lymphocytes, Th1, and Th2. However, these studies had small sample sizes and were not externally validated (Kamat et al., 2018; Martínez et al., 2019; Temiz et al., 2021). No studies have applied medical imaging tests, such as ultrasound, computed tomography (CT), and magnetic resonance imaging (MRI), for predicting BCG treatment response, while they have been widely used for pretreatment prediction of other cancers, such as breast (Liu et al., 2020; Xiong et al., 2021), lung (Liu et al., 2021), and renal cancers (Rallis et al., 2021). Since diagnostic images can depict the phenotypes of bladder cancer in a non-invasive way, recent studies have illustrated that the utilization of imaging biomarkers to predict the response of MIBC with different chemotherapies is feasible (Cha et al., 2017; Hadjiiski et al., 2020; Necchi et al., 2020). Among these non-invasive imaging-based radiomics prediction or classification models, various dimensionality reduction and matrix decomposition methods have been introduced, such as vector quantization and principal component analysis. However, these methods have limited ability to capture the full message of radiomics data from a small region of interest (ROI) in patients with NMIBC, which might account for the fact that no radiomics model has been developed for predicting BCG response in such patients.

Non-negative matrix factorization (NMF), an algorithm based on decomposition by parts (Lee and Sebastian, 1999), has been introduced to identify distinct molecular patterns, while recovering meaningful biological information from tumor-related microarray data (Brunet et al., 2004; Motzer et al., 2020). In this study, we used NMF to decompose the radiomic features from small lesions on contrast-enhanced CT images, which can then be analyzed by combining different features; thus, generating all variabilities of components to represent samples, analogous to gene expression patterns in terms of the metagenes (Brunet et al., 2004). Subsequently, the most relevant component of BCG failures could be selected, which might be a potential biomarker for BCG response.

In this study, we applied NMF and our model selection criterion by factorizing the radiomic features extracted from the pretreatment contrast-enhanced CT images in NMIBC and generated different feature components representing different NMIBC subtypes. We were able to investigate whether radiomics feature components are associated with BCG failure and whether this substaging method can be used to improve patient stratification at diagnosis of NMIBC.

## Materials and Methods

### Study Design

This was a two-center retrospective observational study. An unsupervised factorization algorithm named NMF, which iteratively selects the most robust pattern within pretreatment contrast-enhanced CT images, was proposed to predict the response to BCG in patients with high-risk NMIBC. Histopathological examination after TURBT was performed as per the reference standard. This study was approved by the institutional review boards of West China Hospital and Shang Jin Nan Fu Hospital, and was conducted in accordance with the Declaration of Helsinki, and the requirement for informed consent was waived.

### Patients

Patients who 1) were pathologically diagnosed with HR-NMIBC (Tis or Ta/T1HG urothelial carcinoma) by TURBT; 2) received ≥5/6 BCG induction instillations after TURBT; 3) underwent TURBT or radical cystectomy when a new lesion was found during follow-up cystoscopy; and 4) underwent pretreatment contrast-enhanced CT scanning before TURBT within one to 2 weeks were included in the study. Patients who 1) did not have pre-TURBT pathological results; 2) did not complete BCG induction or had a nonstandard instillation regimen (i.e., the number of BCG instillation less than 5); 3) had confirmative surgery at an external institution, or did not have their recurrence assessed, or had follow-up less than 12 mon; 4) did not undergo pretreatment contrast-enhanced CT scanning; and 5) had insufficient CT quality to obtain measurements (e.g., due to metal artifacts) were excluded from the study.

The primary endpoint of this study was the response status to BCG instillation therapy (BCG failure/BCG response) within 1 year. Specifically, BCG failure was defined according to the European Urology Association guidelines (Babjuk et al., 2019). The secondary endpoint was recurrence-free survival (RFS), defined as the time interval from the beginning of BCG therapy to the first high-grade disease recurrence (BCG failure) during follow-up.

### Image Recognition and Feature Extraction

Contrast-enhanced CT examination of each patient was performed within 1–2 weeks before surgery. CT scanning was performed using a 128-MDCT scanner (SOMATOM Definition Flash, syngo CT 2012B medical system, Siemens, Germany) or a 160-revolution APAX MDCT scanner (Quantix 160 mm × ray cube, GEmedical system, United States). All CT examinations were performed under the following conditions: 120 KVp; 210mA; 14.17 ctdIVOL (mGy); 778.7 DLP (mGy*cm); pitch, 1.0; rotation time, .5 s; section thickness, 2.0 and 5.0 mm.

Three-dimensional region of interest (3D-ROI) was manually delineated on the CT images using ITK-SNAP software (http://www.itksnap.org), and the largest tumor was targeted for patients with multiple lesions in this study. To accurately match the targeted ROI and the pathological result, we had a coordinator to carefully review the surgery records and record the final pathological grades of targeted tumors. Radiologist 1 (4 years' experience) manually drew the 3D-ROIs along the tumor margin, and then the radiologist 2 (10 years' experience) validated these ROIs. To ensure reproducibility of ROIs, intra-class correlation (ICCs) was used for evaluating intra-observer agreement. We randomly selected 30 patients and re-delineated ROIs by radiologists 1 one month later after the initial ROI segmentation. An ICC greater than 0.75 were considered ROIs of satisfactory reproducibility. All images were resampled to a spacing of 1.0*1.0*2.0 cm. We used the image feature extraction software Python package (pyradiomics) to obtain 107 CT-based radiomic features, all of which were based on original images, including 14 shape features, 18 histogram features, and 68 texture features (Supplementary Table S1). All of these features have been previously reported (Aerts et al., 2014; Zhang et al., 2020; Fiz et al., 2021).

### Feature Decomposition and NMF Component Construction

Radiomic features with high collinearity were excluded. Subclasses were then computed by reducing the dimensionality of the expression data from reserved radiomics features to a few meta-features using NMF (Python pakage Nimfa) (Kamat et al., 2018). This method computes multiple k-factor factorization decompositions of the feature matrix, which is the first value where the residual sum of squares curve presents an inflection point (Hutchins et al., 2008).

In traditional matrix decomposition technologies, such as feature decomposition, the decomposed matrix will have negative values, but negative values are meaningless in the actual scene. For example, in the field of image processing, radiomics features are a matrix composed of non-negative numbers, which have no practical significance for the negative values obtained by decomposition processing. Our goal is to find a small number of meta-features, each defined as a positive linear combination of the M radiomics features. Mathematically, this corresponds to factoring matrix V into two matrices with positive entries, V ∼ WH. The shape of V is M × N, M equals to the number of features and N equals to the number of samples, as shown in Figure 2A. Matrix W has size M × k, with each of the k columns defining a meta-feature; entry Wij is the coefficient of feature i in metafeature j. Matrix H has size k × N, with each of the M columns representing the metafeature pattern of the corresponding sample; entry H_ij_ represents the level of metafeature i in sample j. For more convenient expression, we depict the level of the metafeature as the score of this component. As the NMF finds different solutions for different initial conditions, the factorizations were repeated 100 times. To select the metafeature with the most predictive ability for disease relapse, we built single-factor Cox models for each metafeature to find the metafeature with the highest C-index.

### Performance Assessment

The predictive values of the NMF components were evaluated using the receiver operator characteristic and area under curve (AUC). The cutoff values for estimating sensitivity and specificity were determined using the Youden’s index. The prognostic performance of the proposed components was assessed using Harrell’s concordance index (C-index) and Kaplan–Meier log-rank analysis, which was also tested in the validation cohort. Furthermore, confusion matrices were constructed to evaluate the agreement between the observed outcomes and the NMF-predicted outcomes, and a calibration curve was plotted for the evaluation of predicted survival.

### Statistical Analysis Workflow

Descriptive data were summarized as frequencies and percentages. Continuous parametric variables are presented as mean ± standard deviation. Nonparametric variables are shown as mean (interquartile range). Pearson’s chi-square test or Fisher’s exact test was used for categorical variables. Comparisons of continuous variables were conducted using Mann-Whitney U tests or Student’s t-tests. Statistical significance was set at *p* < .05 was considered to be statistically different. Statistical analyses were performed using R software (version 3.8).

## Results

### Clinical Characteristics

**FIGURE 1:**
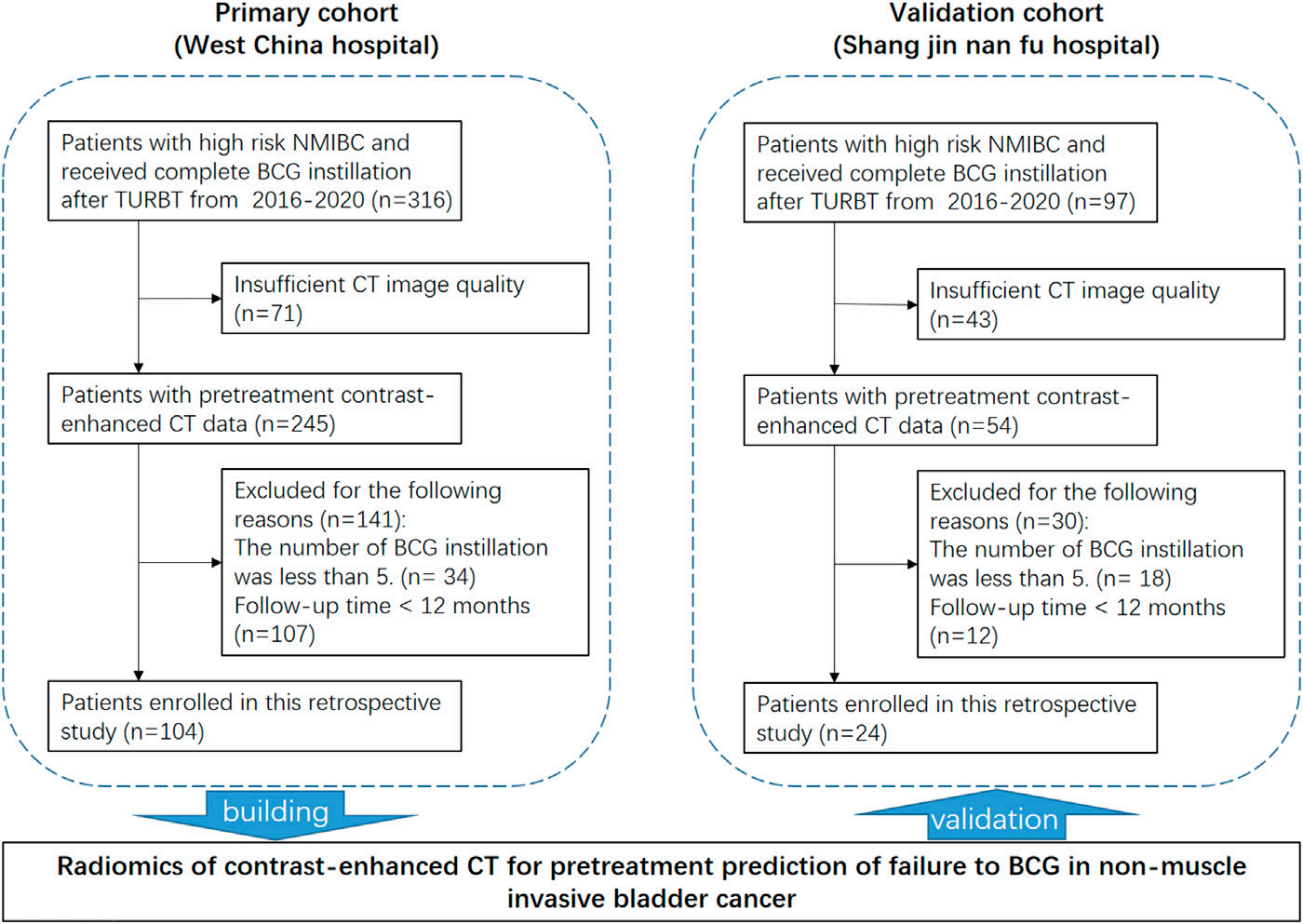
Patient recruitment and study design.

As shown in Figure 1, 413 potentially eligible patients were consecutively retrieved from the databases of two hospitals, and 128 patients were finally included in this study according to the inclusion and exclusion criteria. The dataset from West China Hospital had 108 eligible patients and was used to develop the model. The clinical characteristics of the patients are summarized in Table 1.

**TABLE 1:** Baseline characteristics of the patients in this study.

	Primary cohort (*N* = 104)	Validation cohort (*N* = 24)	*p*
Age (years, mean ± SD)	66.0 ± 11.2	69.2 ± 10.8	.196
Gender			
Male	82 (78.8)	21 (20.2)	0.256
Female	22 (21.2)	3 (79.8)	
Concomitant CIS			.327
No	70 (67.3)	18 (75)	
Yes	34 (32.7)	6 (25)	
Tumor focality			0.522
Unifocal	51 (49.2)	12 (50)	
Multifocal	53 (50.8)	12 (50)	
Tumor size (cm)			0.418
<3	74 (71.2)	16 (66.7)	
≥3	30 (28.8)	8 (33.3)	
Stage			<.001
Ta	58 (55.8)	4 (16.7)	
T1	49 (44.2)	20 (83.3)	
BCG failure			<.001
No	96 (88.9)	13 (54.2)	
Yes	12 (11.1)	11 (45.8)	
Median total BCG instillations (IQR)	19 (19–23)	14 (9—16)	
Median total mos follow-up (IQR)	24 (16–37)	12 (7–21)	
Median mos time to BCG failure (IQR)	9 (8—10)	7 (5–10)	

BCG, *Bacillus* Calmette-Guerin; CIS, carcinoma *in situ*; IQR, inter-quartile range; SD, standard deviation.

The cutoff date of the primary training cohort was June 19, 2021, and the median follow-up time was 24 months (IQR, 16–37 months). Twelve patients (11.1%) had BCG failure. The median RFS was 9 months (IQR, 8–10 months). The cutoff date of the validation cohort was September 30, 2021, and the median follow-up time was 12 months (IQR, 7–21 months). Eleven patients (45.8%) experienced BCG failure. The median RFS was 7 months (IQR, 5–10 months). No significant differences were detected between these two cohorts in terms of age, rate of concomitant carcinoma *in situ*, tumor focality, and size, while the validation cohort had significantly higher proportions of BCG failure (*p* < .001) and T1 stage (*p* < .001).

### Construction of NMF Components

**FIGURE 2:**
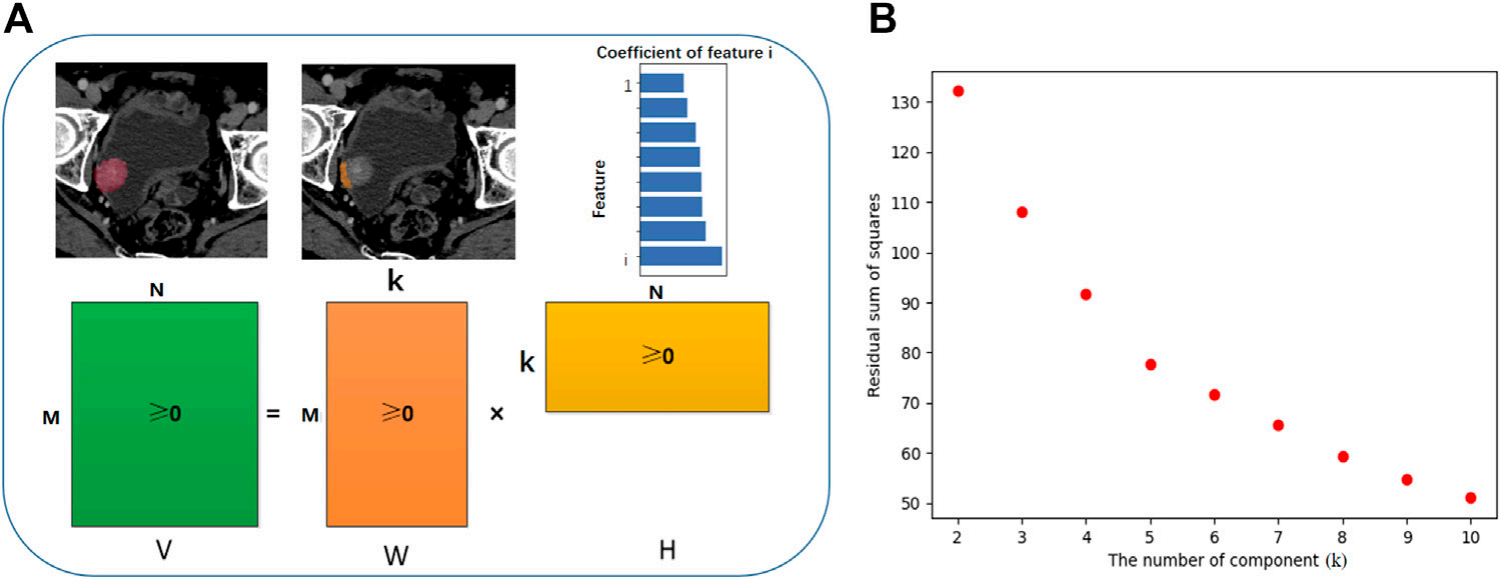
Workflow of non-negative matrix factorization (NMF). **(A)** V represents the original data matrix as the combination of two matrices, V ∼ WH. The shape of V is M × N, M equals to the number of features and N equals to the number of samples. W is a matrix which contains the reduced number of k components derive from NMF, and the sub-classified features for each component (M). Matrix H has size k × N, with each of the M columns representing the metafeature pattern of the corresponding sample. **(B)** The most robust NMF of training cohort selected and tested k = 2 to k = 10, and the turning point was identified as k = 5. That is, NMF identified five components of radiomics features in the primary training cohort.

**FIGURE 3:**
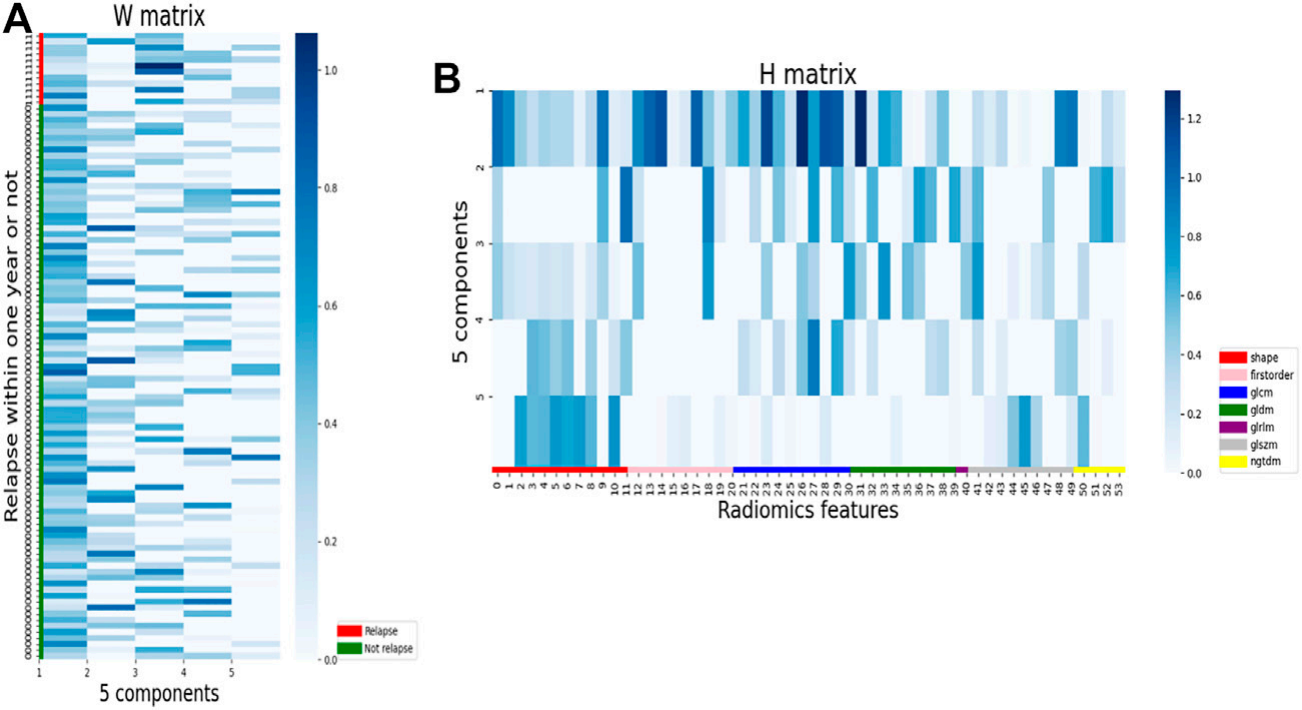
Illustration of component selection with NMF. **(A)** Patients were aggregated by NMF component using the mean across patients for each component, and the mean Z score for each feature was calculated, resulting in one Z score per feature per NMF component. **(B)** Heatmap of radiomics features. Z scores were calculated for each features. Samples are grouped by NMF components.

We excluded 53 radiomic features with high collinearity. To expand our understanding of the radiomics of bladder cancer, we utilized NMF to leverage the CT radiomics dataset in high-risk NMIBC and further identify predictive radiomics biomarkers of BCG failure. The most robust NMF of 108 patients selected and testing k = 2 to k = 10 was identified as k = 5 (Figure 2B). That is, NMF identified five components of radiomics features in the primary training cohort (as shown in the Supplementary Table S2). The W matrix reflects the composition of each component (Figure 3A), and the H matrix reflects the scores of five components for each sample (Figure 3B), from which we can conclude that component 3 is most associated with the failure of BCG treatment, as the level of this component is higher in the samples with the failure of BCG treatment.

### Predictive and Prognostic Performance of NMF Components

**FIGURE 4:**
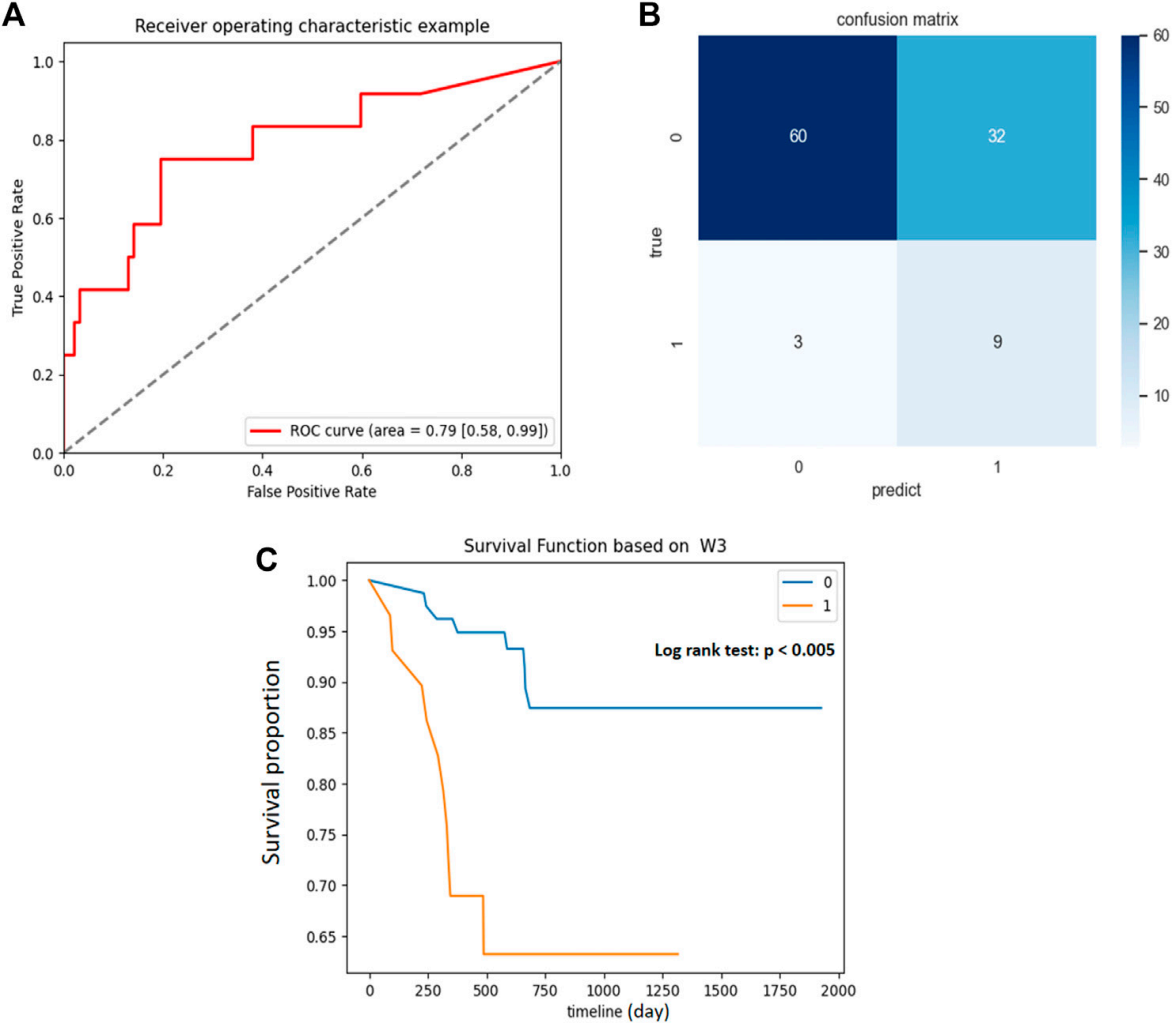
Association between NMF component 3 and clinical outcomes in primary cohort. **(A)** ROC curve and the AUC for the predictive accuracy of NMF component 3 in predicting BCG failure in 1 year. **(B)** Confusion matrix presenting the predictive outcomes using NMF component 3 and true outcomes of BCG failure in 1 year. **(C)** With the component Z score of .2 as the cutoff, patients with scores <.2 (0) had significantly prolonged recurrence free survival (RFS) than those with scores >.2 (1), *p* < .005.

The scores of NMF component 3 yielded a good prediction performance, with an AUC of .79 in the developing cohort (Figure 4A), and accurately predicted 9/12 BCG failures and 60/92 patients without BCG failure (Figure 4B). The optimal cutoff value was the component z-score of .2 with sensitivity and specificity of .75 and .65, respectively. Component 3 showed moderate performance in recurrence-free survival (RFS) estimation in the training cohort, with a C-index of .69. Patients were divided into high-risk and low-risk groups, with a component z-score of .2 as the cutoff. Compared with patients with a z-score of less than .2, patients in the group with z-score larger than .2 had a significantly shorter RFS (Figure 4C, *p* < .005). The associations between the top five features in NMF component 3 and RFS were separately examined, as shown in Supplementary Figure S1.

**FIGURE 5:**
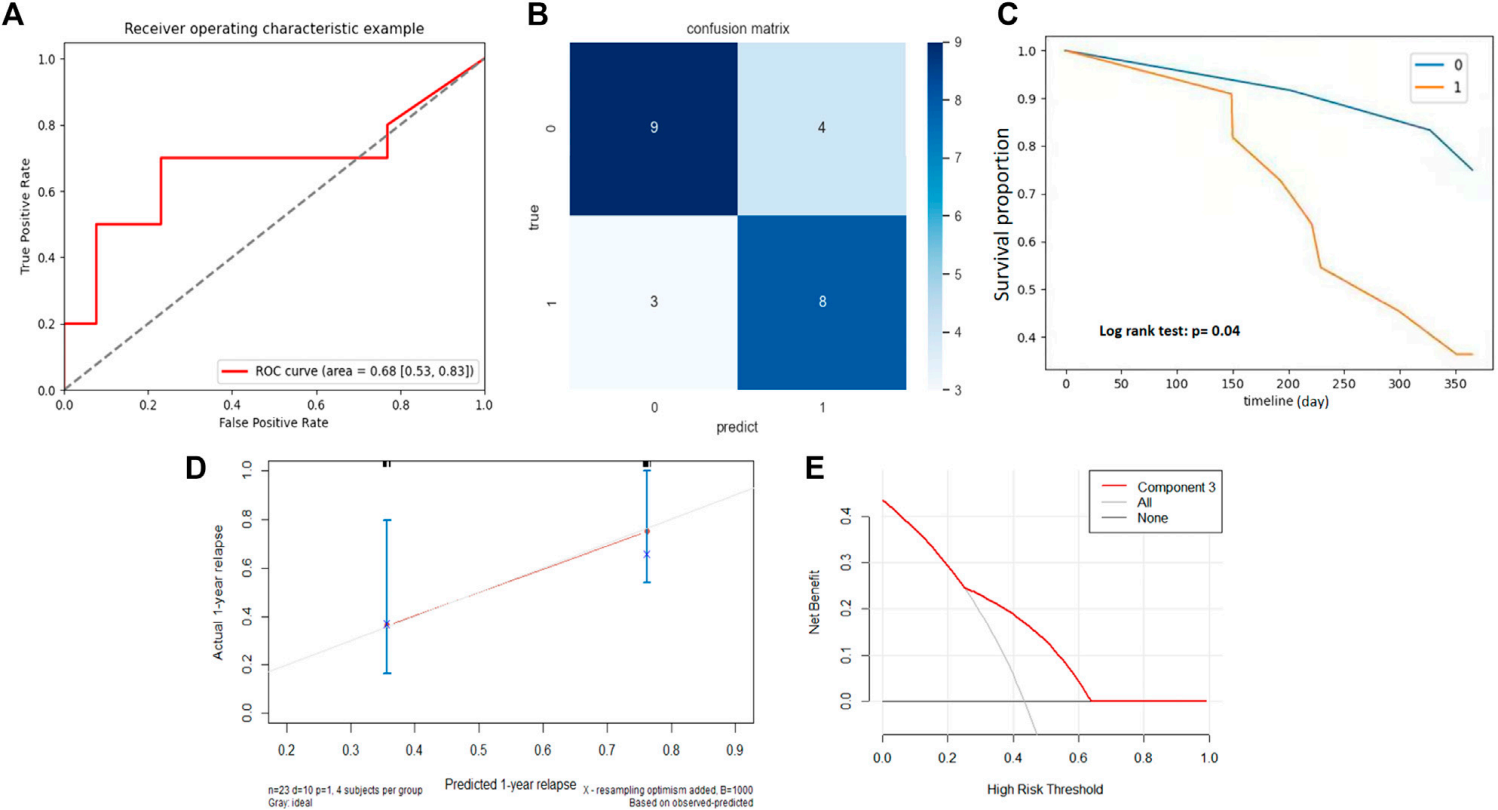
External validation of NMF component 3. **(A)** ROC curve and the AUC for the predictive accuracy of NMF component 3 in predicting BCG failure in 1 year. **(B)** Confusion matrix presenting the predictive outcomes using NMF component 3 and true outcomes of BCG failure in 1 year. **(C)** With the component Z score of .2 as the cutoff, patients with scores <.2 (0) had significantly prolonged recurrence free survival (RFS) than those with scores >.2 (1), *p* = .04. **(D)** Calibration curve of the component 3. **(E)** Decision curve of component 3. The *X*-axis shows the cutoff value, while the *Y*-axis shows the net benefit.

Good performance was also observed for BCG failure prediction in the validation cohort. As shown in Figure 5B, NMF component 3 accurately predicted 8/11 BCG failures and 9/13 patients without BCG failure. Although he AUC of NMF dropped marginally in the validation cohort, the AUC approximated .70 (Figure 5A), and the sensitivity and specificity were .73 and .69, respectively. For prognostic performance, component 3 achieved a moderate performance in the estimation of RFS (C-index, 0.68) in the validation cohort. Compared with patients with a z-score of less than .2, patients in the group with z-score larger than .2 had a significantly shorter RFS (Figure 5C, *p* = .04). The calibration curve and decision curve analysis of the NMF components are shown in Figures 5D,E, which indicate its potential clinical usefulness.

## Discussion

In this two-center study, we investigated the ability of pretreatment contrast-enhanced radiomics analysis so as to predict BCG failure in patients with high-risk NMIBC. An unsupervised strategy named NMF was proposed with better performance in the primary training cohort and performed well in the external validation cohort. The outperformance of NMF indicated that the NMF-decomposed components from CT radiomics features could serve as potential biomarkers for pretreatment predicting BCG failure in patients with high-risk NMIBC.

It is of great guiding significance for the selection of treatment options and clinical decision support of patients with high-risk NMIBC to identify predictive biomarkers related to the BCG response and subsequent recurrence time (Kamat et al., 2018; Ilijazi et al., 2020; Shiota et al., 2020). Currently, most studies have focused on biomarkers in biological specimens, such as peripheral blood, urine, and tumor tissue from surgery. High levels of urine Treg cells and tumor-infiltrating dendritic cells in the pathological examination were associated with rapid recurrence following BCG therapy (Chevalier et al., 2018; Ayari et al., 2009). For prognostic outcome after BCG treatment, Martinez et al. found that patients with a lower T-bet^+^/lymphocyte ratio and higher GTR/NLR had significantly longer recurrence-free survival (Martínez et al., 2019). As to the pathological results, De Jong et al. found that T1 patients with extensive invasion of the lamina propria had a higher risk for BCG failure and an improved progression-free survival (de Jong et al., 2021). However, there are some inherent limitations for these biomarker-finding studies, such as poor specificity of biochemical factors for reacting to both inflammation and tumor, lack of external validation, and high interobserver variability in invasion extension (Babjuk et al., 2019; Del Giudice et al., 2020). The presentation of tumors on radiological images tends to be more stable than biochemistry factors, and image biomarkers extracted from medical images retain excellent stability (Zwanenburg et al., 2020) and are more easily available than pathological substaging. In our study, a non-invasive CT-based NMF component was developed and performed well in an external validation cohort.

Various imaging-based radiomics models have been proposed to predict treatment responses in different cancers (Liu et al., 2021; Rallis et al., 2021;. Zhong et al., 2021) with the hypothesis that these selected imaging features reflect specific tumor phenotypes (Lambin et al., 2012; Aerts et al., 2014). In addition, many other studies have reported the effects of imaging features on survival outcomes, but no studies have been reported regarding BCG instillation on patients diagnosed with high-risk NMIBC. In this study, the proportion of BCG failure in 1 year was too low to construct a traditional radiomics model, which we had tried on, and of which the accuracy was similar to that of flipping a coin. The main reasons for the unexpected low discrimination of traditional radiomics models might be the low proportion of BCG failure and relatively greater amount of radiomic features, which increased the difficulty of traditional machine learning methods to discover patterns of BCG failure cases (van der Ploeg et al., 2014; Gillies, Kinahan, and Hricak 2016; Moons et al., 2019). The goal of our research is similar to gene expression studies, of which a handful metagenes are selected from thousands of genes in limited samples. This can be achieved with NMF, which is an unsupervised algorithm based on decomposition by parts and a model selection mechanism. NMF has been used to iteratively select the most robust biomarkers from thousands of genes (Zhong et al., 2018; Zeng et al., 2019; Motzer et al., 2020) and to find structural covariance patterns in neuroimaging content (Nassar et al., 2019; Patel et al., 2020). In our application of NMF to radiomic features, the parts were the components of a reduced representation of the original hidden features, which may enable the recovery of biologically similar phenotypes. Considering that the molecular mode of BCG actions remains partially understood, NMF components might be a hint for potential pathways for BCG treatment failure based on previous reports about the cellular geography associated with the poor response to BCG (Ilijazi et al., 2020; Shiota et al., 2020; Tran et al., 2020).

Compared with CT, the superiority of MRI has been documented with respect to the diagnostic performance and evaluation of treatment response of BCa, but there have been no studies on their performance in radiomics analysis (Wong et al., 2021). For MRI-based radiomics, it is quite difficult to standardize image acquisitions for numerous parameters and many variations among manufacturers with different magnetic fields (Wakabayashi et al., 2019). In addition, MRI is susceptible to many artifacts, such as image and signal distortion consequences due to contiguous gas-filled bowls and gas bubbles within the bladder (Lin and Chen, 2015) which could complicate the reproducibility of measurements. In studies on CT radiomics, automatic acquisition protocol and test-retest analysis have proven to be useful in overcoming the bias of acquisition protocols (Caruso et al., 2021). In light of the above limitations, radiomic models based on MRI are more difficult to reproduce across institutions than those based on CT images (Harding-Theobald et al., 2021). CT is recommended prior to TURBT according to the NCCN guidelines, and is still the most commonly used imaging method worldwide in diagnosing and staging BCa, mainly because CT is fast and inexpensive (Babjuk et al., 2019; Flaig et al., 2020). Considering the easily acquired CT images across hospitals, the CT radiomics model can be clinically validated on a larger sample. As expected, the NMF components from CT radiomics demonstrated a stable performance in this double-center study, and further large-scale studies are needed to determine the reproducibility and clinical utility of NMF components.

Despite these remarkable results, our study has several inherent limitations. First, although we found a robust component, which was strongly related to the risk of BCG failure in 1 year and associated with the recurrence survival after BCG instillation, we failed to uncover the underlying molecular mechanisms of these nested radiomic features. Further investigation could focus on the comparison of gene/molecular expression patterns among different subtypes defined by radiomic features. Second, owing to the retrospective design of this study, some inherent limitations were inevitable, such as the high proportion of excluded cases for missing data and those with poor quality in CT images. Besides, the retrospective design might be the main cause of inconsistent recurrence rates in the two cohorts. Given the fact that our NMF strategy performed well in both cohorts with extremely different BCG failure rates, we still have confidence in the further predictive validation of NMF strategy. Future studies should enroll more patients with standard BCG instillation with regular follow-up, so that the NMF strategy could be better validated.

In conclusion, the present preliminary study suggests that NMF could provide a potential tool for predicting BCG response and survival outcomes in patients with high-risk NMIBC. With further molecular research, NMF-based components may be useful as molecular biomarkers of treatment response.

## Data Availability

The raw data supporting the conclusion of this article will be made available by the authors, without undue reservation.
