# Supplementary material for: Radiomics of Contrast-Enhanced Computed Tomography: A Potential Biomarker for Pretreatment Prediction of the Response to Bacillus Calmette-Guerin Immunotherapy in Non-Muscle-Invasive Bladder Cancer
Source: Front Cell Dev Biol. 2022 Feb 25;10:814388. doi: 10.3389/fcell.2022.814388 (PMC8914064; doi:10.3389/fcell.2022.814388)
Supplement: Supplementary file 2 [file Table1.DOCX]

**Supplementary table 1. 107 radiomics features extracted from the original contrast-enhanced CT images.**

| original_shape_Elongation |
| --- |
| original_shape_Flatness |
| original_shape_Leas0xisLength |
| original_shape_MajorAxisLength |
| original_shape_Maximum2DDiameterColumn |
| original_shape_Maximum2DDiameterRow |
| original_shape_Maximum2DDiameterSlice |
| original_shape_Maximum3DDiameter |
| original_shape_MeshVolume |
| original_shape_MinorAxisLength |
| original_shape_Sphericity |
| original_shape_SurfaceArea |
| original_shape_SurfaceVolumeRatio |
| original_shape_VoxelVolume |
| original_firstorder_10Percentile |
| original_firstorder_90Percentile |
| original_firstorder_Energy |
| original_firstorder_Entropy |
| original_firstorder_InterquartileRange |
| original_firstorder_Kurtosis |
| original_firstorder_Maximum |
| original_firstorder_MeanAbsoluteDeviation |
| original_firstorder_Mean |
| original_firstorder_Median |
| original_firstorder_Minimum |
| original_firstorder_Range |
| original_firstorder_RobustMeanAbsoluteDeviation |
| original_firstorder_RootMeanSquared |
| original_firstorder_Skewness |
| original_firstorder_To0lEnergy |
| original_firstorder_Uniformity |
| original_firstorder_Variance |
| original_glcm_Autocorrelation |
| original_glcm_ClusterProminence |
| original_glcm_ClusterShade |
| original_glcm_ClusterTendency |
| original_glcm_Contrast |
| original_glcm_Correlation |
| original_glcm_DifferenceAverage |
| original_glcm_DifferenceEntropy |
| original_glcm_DifferenceVariance |
| original_glcm_Id |
| original_glcm_Idm |
| original_glcm_Idmn |
| original_glcm_Idn |
| original_glcm_Imc1 |
| original_glcm_Imc2 |
| original_glcm_InverseVariance |
| original_glcm_Join0verage |
| original_glcm_JointEnergy |
| original_glcm_JointEntropy |
| original_glcm_MCC |
| original_glcm_MaximumProbability |
| original_glcm_SumAverage |
| original_glcm_SumEntropy |
| original_glcm_SumSquares |
| original_glrlm_GrayLevelNonUniformity |
| original_glrlm_GrayLevelNonUniformityNormalized |
| original_glrlm_GrayLevelVariance |
| original_glrlm_HighGrayLevelRunEmphasis |
| original_glrlm_LongRunEmphasis |
| original_glrlm_LongRunHighGrayLevelEmphasis |
| original_glrlm_LongRunLowGrayLevelEmphasis |
| original_glrlm_LowGrayLevelRunEmphasis |
| original_glrlm_RunEntropy |
| original_glrlm_RunLengthNonUniformity |
| original_glrlm_RunLengthNonUniformityNormalized |
| original_glrlm_RunPercen0ge |
| original_glrlm_RunVariance |
| original_glrlm_ShortRunEmphasis |
| original_glrlm_ShortRunHighGrayLevelEmphasis |
| original_glrlm_ShortRunLowGrayLevelEmphasis |
| original_glszm_GrayLevelNonUniformity |
| original_glszm_GrayLevelNonUniformityNormalized |
| original_glszm_GrayLevelVariance |
| original_glszm_HighGrayLevelZoneEmphasis |
| original_glszm_LargeAreaEmphasis |
| original_glszm_LargeAreaHighGrayLevelEmphasis |
| original_glszm_LargeAreaLowGrayLevelEmphasis |
| original_glszm_LowGrayLevelZoneEmphasis |
| original_glszm_SizeZoneNonUniformity |
| original_glszm_SizeZoneNonUniformityNormalized |
| original_glszm_SmallAreaEmphasis |
| original_glszm_SmallAreaHighGrayLevelEmphasis |
| original_glszm_SmallAreaLowGrayLevelEmphasis |
| original_glszm_ZoneEntropy |
| original_glszm_ZonePercen0ge |
| original_glszm_ZoneVariance |
| original_gldm_DependenceEntropy |
| original_gldm_DependenceNonUniformity |
| original_gldm_DependenceNonUniformityNormalized |
| original_gldm_DependenceVariance |
| original_gldm_GrayLevelNonUniformity |
| original_gldm_GrayLevelVariance |
| original_gldm_HighGrayLevelEmphasis |
| original_gldm_LargeDependenceEmphasis |
| original_gldm_LargeDependenceHighGrayLevelEmphasis |
| original_gldm_LargeDependenceLowGrayLevelEmphasis |
| original_gldm_LowGrayLevelEmphasis |
| original_gldm_SmallDependenceEmphasis |
| original_gldm_SmallDependenceHighGrayLevelEmphasis |
| original_gldm_SmallDependenceLowGrayLevelEmphasis |
| original_ngtdm_Busyness |
| original_ngtdm_Coarseness |
| original_ngtdm_Complexity |
| original_ngtdm_Contrast |
| original_ngtdm_Strength |
