# Supplementary figures and images for: Radiomics of Contrast-Enhanced Computed Tomography: A Potential Biomarker for Pretreatment Prediction of the Response to Bacillus Calmette-Guerin Immunotherapy in Non-Muscle-Invasive Bladder Cancer
Source: Front Cell Dev Biol. 2022 Feb 25;10:814388. doi: 10.3389/fcell.2022.814388 (PMC8914064; doi:10.3389/fcell.2022.814388)

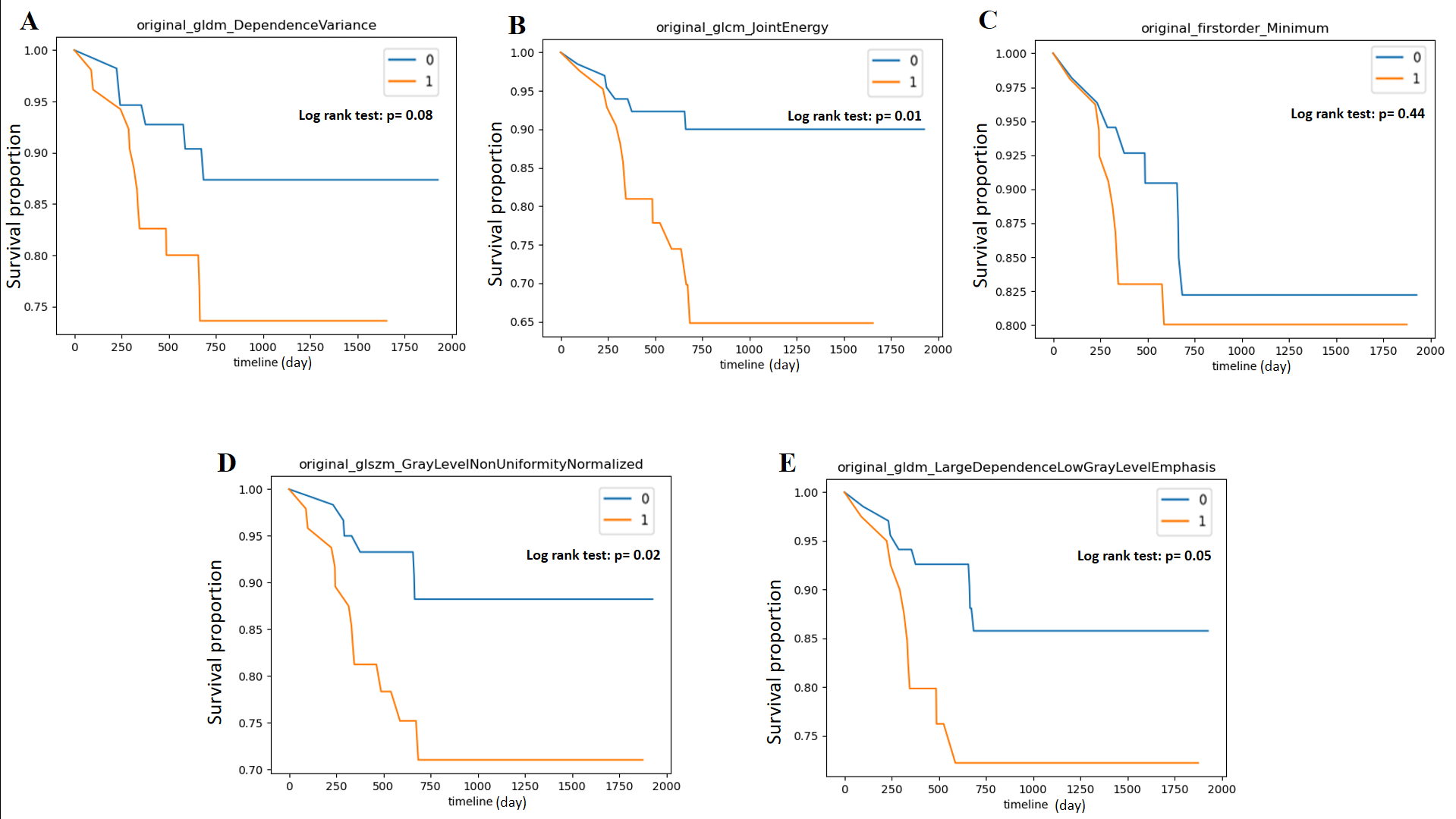

Supplement: Supplementary file 3 [file Image1.tif]
